# Non-adherence to antihypertensive medications in Lebanese adults hospitalized for hypertensive urgency and its cost

**DOI:** 10.1186/s12872-022-02907-z

**Published:** 2022-11-01

**Authors:** Hanine Abbas, Souheil Hallit, Mazen Kurdi, Rita Karam

**Affiliations:** 1 Department of chemistry and Biochemistry, Faculty of Science, Lebanese University, Beirut, Lebanon; 2 School of Medicine and Medical Sciences, Holy Spirit University of Kaslik, P.O Box 446, Jounieh, Lebanon; 3 Research Department, Psychiatric Hospital of the Cross, Jal Eddib, Lebanon; 4 Quality Assurance of Pharmaceutical Products Department, Lebanese Ministry of Public health, Baabda, Lebanon; 5 Pharmacology Department, Faculty of Medical sciences, Lebanese University, Beirut, Lebanon

**Keywords:** Hypertensive urgency, Non-adherence, Hospitalization cost, Ministry of Public Health, Lebanon

## Abstract

**Background:**

Drug non-adherence is assumed to play an important role in development of hypertensive urgency, which is a common health problem resulting in frequent emergency department admissions and thus increased healthcare spending wastage. The objective of this study is to assess the rate of non-adherence to antihypertensives and to evaluate influencing factors predicting this behavior in Lebanese hypertensive adults. In addition, this study aim to estimate the cost of hospitalization for hypertensive urgency covered by the Ministry of Public Health in patients’ non-adherent to their antihypertensives.

**Methods:**

A multi-methods approach is used comprising a cross-sectional study, additionally to an observational, retrospective, cost of illness study. A cross-sectional questionnaire based study is conducted from May to Dec, 2019 to address the study objective. Using the Ministry of Public Health hospitalization data during 2019, the cost of hospitalization for hypertensive urgency is assessed. Multivariable analysis is performed to calculate the adjusted odd ratios by fitting a logistic regression model.

**Results:**

The cross-sectional study includes 494 participants and shows that 43.0% of patients hospitalized and covered by the Ministry of Public Health are non-adherent. The univariate regression model shows that adherence to antihypertensive medications is significantly associated with age (*p*-value = 0.005) and follow-up visits (p-value = 0.046). The odds of adherence for participants earning more than USD 2000 was 3.27 times that for those who earn less than USD 1000 (*p* = 0.026). The estimated cost of hospitalization for non-adherent patients is USD 452,353 in 2019.

**Conclusion:**

Non-adherence associated hospitalization costs represents a financial burden to Lebanese health system.

## Background

Hypertensive crises, defined as systolic blood pressure (BP) greater than 180 mmHg or diastolic BP greater than 120 mmHg, are responsible for nearly 8 million hospitalizations each year and 20 million visits to the emergency department [1]. They may be further categorized as hypertensive emergencies or urgencies. Hypertensive emergencies are distinguished by evidence of progressive target organ dysfunction. While in hypertensive urgencies (HUs), there are no evidence of target organ damage; such as pulmonary edema, cardiac ischemia, neurologic deficits, or acute renal failure [1]. Non-adherence to medications is presumed to play a critical role in development of hypertensive crises and fueling the public health crisis of ineffectually treated hypertension (HT) [2]. The metric of “adherence” simply apprehended concordance of patient’s self-management behavior with alternately agreed health care directions [2]. Unfortunately, there is bountiful evidence to show that medications adherence is frequently suboptimal and this originates a notable cost in terms of forgone clinical and economic benefit [2]. The 2013 Intercontinental Marketing Statistics (IMS) “Avoidable Costs in US Healthcare” report specifies that annually USD 105.4 billion would be preventable if medication non-adherence was tackled [3]. Additionally, based on a U.S. study in 2019, if the 25% of Medicare beneficiaries with HT who were non-adherent became adherent, Medicare could save USD 13.7 billion annually, with over 100,000 emergency department visits and 7 million inpatient hospital days that could be prevented [4]. In Australia, it is estimated that USD 6 billion annually is avertible due to poor medicines usage [5]. A systematic review disclosed the annual adjusted disease-specific economic cost of medications non-adherence to range from USD 949 to USD 44,190 per person and the corresponding costs of non-adherence to cardiovascular diseases therapies (CVDs) were USD 16,124 per person [2].

Hospitalization costs are the main driver of healthcare spending inflation. Noticeably, inappropriate use of medicines costs the Australian public hospital system USD 900 million per year representing 2–3% of all hospital admissions, with this figure rising to 20–30% of all admissions in the population aged 65 years and over [5]. Moreover, other studies also showed a higher risk of hospitalization with poor adherence to antihypertensive medications and this risk decreased with increasing adherence [6].

In Lebanon, hospitalization accounts for 40% of total health expenditure. The total annual health expenditures per capita reached USD 930 in 2017, totaling USD 3.8 billion, accounting for 7.4% percent of the Gross Domestic Product (GDP) according to published statistics by the Ministry of Public Health (MoPH) [7]. Lebanon is characterized by a highly fragmented healthcare system. The MoPH provides health care services free of charge to almost half of the population. The other half of the population is covered by National Social Security Funds (NSSF) (28%), military schemes (5%), Civil Servants Cooperative (CSC) (5%) and finally the private insurance and mutual funds cover the remaining part (12%) (7). According to the latest statistical bulletin, the MoPH budget per capita reached USD 106 in 2017, a 16% increase from 2016. The MoPH’s budget grew to USD 486 million, a 14% increase from 2016 [7]. In addition, the MoPH is the main financier for hospitalization, allocating about 64% of its total annual budget for hospitalization coverage, which was about 309 million USD in 2018 [7].

In Lebanon, while there is a substantial information relating non-adherence to poor patient outcomes, to our knowledge, investigations into economic impact of non-adherence to antihypertensives are lacking. Due to the scarcity of healthcare resources, estimating hospitalization cost associated with the non-adherence to medications enables greater planning in terms of health policy to help address increasing and avoidable costs. In light of the above, the aim of this study is to assess the rate of non-adherence to antihypertensive medications and to evaluate influencing factors predicting this behavior in Lebanese hypertensive adults. In addition, this study points to estimate the cost of hospitalization for HU covered by the MoPH in non-adherent hypertensive patients using a multi-methods approach.

## Methods

Due to the lack of direct published evidence on the rate of non-adherence among hypertensive patients covered by MoPH, a multi-methods approach is used, in which two research methods are included; the first one comprise to a cross-sectional study, in addition to an observational, retrospective, cost of illness study. Following separate analyses, results of both studies are triangulated to address the objectives of this work.

### Cross-sectional study of adherence to antihypertensive medications

#### Study design

A questionnaire-based cross-sectional study is conducted from May to December 2019 to address the study objective. Participants are randomly selected from outpatients’ clinics in Rafic-Hariri University hospital and community pharmacies distributed in two densely populated Lebanese regions Beirut and Mount Lebanon (called Greater Beirut Area), with around 50% of inhabitants living there. Questionnaires are administered by trained pharmacists via face-to-face interviews that lasted up to 30 minutes. Arabic, the country’s official language, is used during interviews.

#### Ethics approval

The study protocol is reviewed and approved by the IRB of Rafic-Hariri University hospital. In accordance with the declaration of Helsinki, all patients who participated in the study give a written informed consent.

#### Cross-sectional study participants

Eligible patients are selected by the pharmacists conducting the study and counseled regarding the study objective. All patients who consented to participate are recruited. Eligible patients are Lebanese adult patients 18 year of age and above. They are diagnosed with essential HT and taking at least one antihypertensive medication such as Diuretics, Beta-blockers, ACE inhibitors, Angiotensin II receptor blockers and Calcium channel blockers. Excluded subjects are pregnant women, subjects with mental disabilities, patients with other CVDs and non-Lebanese subjects.

#### Non-adherence measurement tool

Non-adherence to antihypertensive medications is self-reported. Participants are asked to indicate whether they take their antihypertensive medications as prescribed by their healthcare providers or not. To validate the participants’ answers, the Drug Attitude Inventory (DAI) scale are used. The DAI scale includes a series of 10 questions each with true/false answers, pertaining to various aspects of the patient’s perceptions and experiences of treatment. Participants are asked to answer each statement in the questionnaire. Each “positive” answer is given a score of plus one, and each “negative” answer is given a score of minus one. A positive total score indicates a positive subjective response (adherent) and a negative total score indicates a negative subjective response (non-adherent).

Thus, non-adherent patients are classified as patients who directly admitted their non-adherence to antihypertensive medications and simultaneously have a negative DAI score while adherent patients are the patients who directly admitted their adherence to antihypertensive medications and simultaneously have a positive DAI score. Patients who their self-reported adherence disagree with DAI score result are excluded.

#### Health insurance and hospitalization in non-adherent patients

Participants are asked to specify the responsible party for their insurance. Hospitalization data is acquired via self-reported questionnaire as participants are asked if they are hospitalized in 2019 due to HU. Hospitalized patients who are classified as non-adherent and covered by the MOPH are selected.

#### Statistical analysis

The data on 494 questionnaires is entered and analyzed by Statistical Package for the Social Sciences (SPSS) program version 21. Descriptive statistics are generated to examine the distribution of the sample according to main variables and covariates, presenting the frequencies and percentages for the binary and categorical variables. Valid percentages are reported. The bivariate associations between the covariates and the antihypertensive medication adherence is presented using unadjusted crude Odds Ratios (ORs) and 95% confidence intervals. Multivariable analysis is performed to calculate the adjusted ORs by fitting a logistic regression model with the outcome measuring antihypertensive medication adherence controlling for potential confounders. The overall level of significance is taken as 5% (*p*-value< 0.05) and 95% confidence intervals are computed for all ORs.

### Cost of illness study

Data on Lebanese hypertensive patients hospitalized for HUs are collected from the database of MoPH. A letter is sent to the general director of the MoPH to get permission for accessing the database and starting the data collection. The search include the hospital information system of the MoPH from the first of January to the end of December 2019. The MoPH comprises utilization data from a total of 163 hospitals distributed all over Lebanon. Data collected include information on admission and discharge dates, services rendered, and charges. Adult patients (⩾18 years) admitted to hospitals for HU are included; the latter is defined by the American Heart Association (AHA) as systolic BP greater than 180 mmHg and/or diastolic BP greater than 120 mmHg without evidence of target organ damage. The billing data related to the selected patients is used to estimate hospitalization costs among Lebanese hypertensive population hospitalized for HUs. Costs are calculated according to the quantity of resources consumed by each patient from admission until discharge from hospital. The bills for each patient are provided by the hospitals’ administration to MoPH including information related to cost of hospitalization (room and boards), laboratory, general exams and cardiology-related investigations, pharmacy cost (medication, serum, and paramedic supplies), nursing charges, and physicians fees. Total direct in-hospital cost for all selected hospitalized patients is calculated. In addition, an average cost per one hospitalized patient is presented as the median value of the total hospitalization cost. Costs calculated in Lebanese Pound (LBP) are converted to USD and they were adjusted to 2019 USD by purchasing power parities and consumer prices index. (Exchange rate: USD1 = LBP 1508) [8]. Data such as nights of hospital stay was presented as means ± SDs. The analysis was conducted with de-identified data to ensure protection of confidential information.

### Hospitalization cost of non-adherent patients covered by the MOPH

In order to estimate the total hospitalization cost for HU in non-adherent patients covered by the MoPH in Lebanon, the number of non-adherent hospitalized patients covered by the MoPH (N) is estimated. The percentage of non-adherent hospitalized patients for HU covered by the MoPH calculated from the cross-sectional study is used to estimate the number of non-adherent patients of the total hospitalized population extracted from the MoPH data in the observational, retrospective, cost of illness study. In addition, the average cost of hospitalization for HU per patient calculated in this last study (observational, retrospective, cost of illness) is used.

## Results

### Participants characteristics in cross-sectional study

The analyses are based on 494 patients who met the inclusion criteria. About three-quarters (73.1%) of the sampled participants are recruited from Rafic-Hariri University hospital. The sample is equally distributed between males and females. About 82.0% of participants are married and 51.6% are unemployed. Only 39.9% of Participants had health coverage while the rest rely on MoPH coverage. Additionally, 28.7% of the patients are hospitalized in 2019 for HU. (Table 1).

**Table 1:** Demographic, Socio-Economic Characteristics of Study Participants (*N* = 494)

Variable			N (Percentage)
**Location**	Community Pharmacy		133 (26.9)
	Hospital		361 (73.1)
**Age**	18–25		3 (0.6)
	26–45		46 (9.3)
	46–65		244 (49.4)
	66 or older		201 (40.7)
**Occupation**	Student		5 (1.0)
	Retired		63 (12.8)
	Employee		171 (34.6)
	Not Employed		255 (51.6)
**Marital Status**	Single		62 (12.6)
	Married		406 (82.2)
	Divorced		11 (2.2)
	Widowed		15 (3.0)
**Hospitalized for HU in 2019**	Never		352 (71.3%)
	Hospitalized		142 (28.7%)
**Income**	Less than 1000USD		393 (79.6)
	1000 to 2000USD		61 (12.3)
	More than 2000USD		40 (8.1)
**Education**	Illiteracy		125 (25.3)
	Primary education		139 (28.1)
	Secondary education		101 (20.4)
	University studies		129 (26.1)
**Health Coverage**	Yes	NSSF	149 (30.2)
		COOP	5 (1.0)
		SF	2 (0.4)
		MOPH	297 (60.1)
		Private insurance	41 (8.3)

### Antihypertensive medications adherence measures

**Fig. 1:**
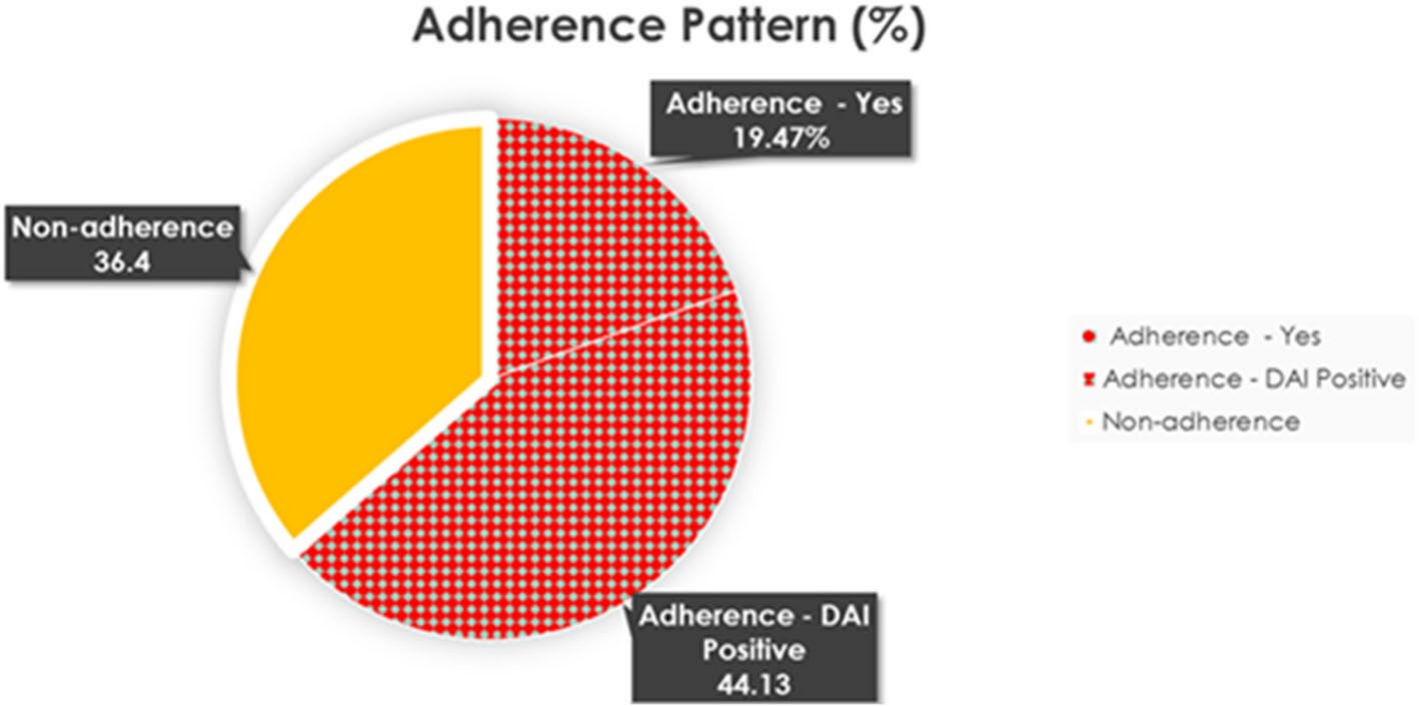
Adherence Pattern. Dotted area represents the 63.6% who are considered to be adherent to medications after merging the results of the DAI and the self-reported adherence to prescribed medications while the rest is considered non adherent

As seen in Tables 2, 69.4% report taking their medications as prescribed by their doctors while 26.3% do not take them because of simple forgetfulness. The vast majority (90.1%) of participants have a positive score on the DAI, indicating a positive subjective adherence response. When merging the results of the DAI and the self-reported adherence to prescribed medications, 63.6% are found to be adherent to medications while 36.4% are found to be non-adherent to their antihypertensive medications (Fig. 1).

**Table 2:** Adherence Pattern of Hypertensive Patients (*N* = 494)

Variables			N (Percentage)
**Self-Reported Adherence Question**	Yes, adherent		343 (69.4)
	No, non- adherent	Side Effects	1 (0.2)
		Simple Forgetfulness	130 (26.3)
		Complexity	11 (2.3)
		Don’t need it/don’t think it works	9 (1.8)
**Drug Attitude Score**	Positive Score (Adherent)		445 (90.1)
	Negative Score (Non-adherent)		49 (9.9)

### Factors associated with antihypertensive medication adherence

The factors associated with antihypertensive medication adherence of study participants are presented in Tables 3 and 4, respectively. The univariate regression model shows that adherence to antihypertensive medications is significantly associated with age (*p*-value = 0.005) and follow-up visits (p-value = 0.046). Being aged between 46 and 65 years (OR = 2.03) or 66 years and older (OR = 2.82) is significantly associated with higher odds of adherence to medications compared to those aged between 18 and 45 years.

**Table 3:** Variables Associated with Antihypertensive Medication Adherence (*N* = 494)

Variable	Number of Adherent Patients (***N*** = 314) N (Percentage)		Crude OR	95% CI	p-Value
**Age**					
18–45 (ref)	22	(7.0)	–	–	0.005
46–65	152	(48.4)	2.03	[1.09–3.77]	0.025
66 or older	140	(44.6)	2.82	[1.49–5.33]	0.001
**Gender**					
Male (ref)	163	(51.9)	–	–	–
Female	151	(48.1)	0.81	[0.56–1.17]	0.262
**Occupation**					
Student (ref)	3	(1.0)	–	–	0.545
Retired	45	(14.3)	1.67	[0.26–10.82]	0.593
Employee	105	(33.4)	1.06	[0.17–6.52]	0.949
Not Employed	161	(51.3)	1.14	[0.19–6.96]	0.886
**Income**					
Less than 1000USD (ref)	246	(78.3)	–	–	0.136
1000 to 2000USD	37	(11.8)	0.92	[0.53–1.60]	0.771
More than 2000USD	31	(9.9)	2.06	[0.95–4.44]	0.066
**Follow-up**					
No regular checkups (ref)	94	(29.9)	–	–	0.046
Monthly	74	(23.6)	1.89	[1.12–3.19]	0.017
Every 3–6 Months	126	(40.1)	1.56	[1.01–2.40]	0.045
Once a year	20	(6.4)	0.96	[0.46–1.98]	0.906

**Table 4:** Multivariable Logistic Regression Models between Antihypertensive Medication Adherence and Associated Variables (N = 494)

Variable	Number of Adherent Patients (***N*** = 314) N (Percentage)		Adjusted OR	95% CI	p-Value
**Age**					
18–45 (ref)	22	(7.0)	–	–	–
46–65	152	(48.4)	2.14	[1.07–4.28]	0.031
66 or older	140	(44.6)	3.67	[1.76–7.64]	0.001
**Gender**					
Male (ref)	163	(51.9)	–	–	–
Female	151	(48.1)	0.96	[0.61–1.51]	0.863
**Occupation**					
Student (ref)	3	(1.0)	–	–	–
Retired	45	(14.3)	1.04	[0.13–8.17]	0.973
Employee	105	(33.4)	0.54	[0.07–4.10]	0.548
Not Employed	161	(51.3)	0.88	[0.12–6.57]	0.903
**Income**					
Less than 1000USD (ref)	246	(78.3)	–	–	–
1000 to 2000USD	37	(11.8)	0.99	[0.49–2.01]	0.986
More than 2000USD	31	(9.9)	3.27	[1.16–9.24]	0.026
**Follow-up**					
No regular checkups (ref)	94	(29.9)	–	–	–
Monthly	74	(23.6)	2.03	[1.12–3.68]	0.020
Every 3–6 Months	126	(40.1)	1.58	[0.99–2.54]	0.056
Once a year	20	(6.4)	0.81	[0.37–1.77]	0.598

Adjusting for all other variables such as age, gender, marital status, occupation, educational level, income, follow up visits, last BP reading and health coverage, the multiple logistic regression model revealed that adherence to medications is significantly associated with age, income, and follow-up visits. Being aged between 46 and 65 years (OR = 2.14) or 66 years and older (OR = 3.67) compared to those aged between 18 and 45 years, doing monthly follow-ups (OR = 2.03) compared to never, and having a monthly income of more than 2000 USD (OR = 3.27) compared to less than 1000 USD are significantly associated with higher odds of adherence to medications.

### Observational, retrospective, cost of illness study

A total of 487 hypertensive patients with no other cardiovascular co-morbidities are admitted to different hospitals across Lebanon for HU with an average of 2 ± 0.42 nights hospital stay. The direct in-hospital cost for all these cases provided by the database of the MoPH is USD 1,054,046 for a total stay of 974 days (USD 1082 ± 15,663 per in-hospital day). The average in-hospital cost per one case is USD 2164. Of the total cost, 26.8% is attributed to the cost of room and board, 20.6% to pharmacy (drugs, serum, and paramedical supplies), 17.3% to laboratory tests, 16.7% to physicians’ fees, 14.4% to general exams (including vascular imaging and cardiology-related investigations), and 4.2% to other expenses (respiratory therapy, and radiology).

### Multi-method approach: estimated hospitalization cost of non-adherent patients covered by MOPH in 2019

The cross-sectional study shows that 43.0% of patients hospitalized and covered by the MoPH are non-adherent while 57.0% are adherent. Assuming that 43.0% of the total hospitalized population extracted from the Cost of Illness Study (*N* = 487) are non-adherent, the estimated number of non-adherent Lebanese hospitalized patients for HUs covered by the MoPH in 2019 is 209 patients. As the average cost of hospitalization for HU per one patient is USD 2164, the estimated cost of non-adherent patients hospitalized for HU is USD 452,353.

## Discussion

To the best of our knowledge, this is the first study estimating the direct in hospital cost due to HU of non-adherent patients in Lebanon. In our cross-sectional study, the overall non-adherence percentage is 36.4%. Our findings echo the percentage of non-adherence to antihypertensive therapy in Europeans (36.6%) and Americans (36.6%) regions according to a meta-analysis published in 2017 [9]. While higher percentage of poor adherence levels has been shown in African patients (62.4%) and Asians (43.5%) regions [9]. Notably, the proportion of adherent patients is modest, illustrating a potential for improvement. Lebanon spends large amount of its health expenditures on patients hospitalized for HUs. The average in-hospital cost for HU per patients was USD 2164. Although a direct comparison is not possible, mean hospital cost per hypertensive patient was close to that reported from USA: (USD 2254) [10] and Canada: (USD 2341) [11]. Yet it was higher than figures reported from Zimbabwe: (USD 1124) [12], Vietnam: (USD 65) [13] and Philippines: (USD 57) [14]. All costs were adjusted to 2019 USD by purchasing power parities and consumer prices index. Due to the lack of direct published evidence on the rates of non-adherence among patients covered by MoPH, we relied on the rate of non-adherence among our cross-sectional study participants covered by the MoPH and who were hospitalized for HU in 2019. Combining this result with the results of our observational, retrospective, cost of illness study, makes the assumption that approximately 43.1% of the hospitalized population for HU covered by MoPH was non-adherent to their antihypertensive medications with an estimated in-hospital cost of USD 452,353. This cost burden can be avoided or decreased if adherence to medication improves. In accordance with our results, Overgauw et al. showed that one-third of patients admitted to emergency department for HU was non-adherent [1]. Another study showed that drug non-adherence was recorded as an attributing factor to HU [2]. A cohort study revealed that among hospitalized patients, poor adherence to antihypertensive medications was linked with increased costs by around USD 3574 (95% CI, USD 2897–USD 4249) per person within a 3-year period [15]. In addition, of all medication-related hospitalizations that happen in the United States, between one-third and two-thirds are the result of poor medication adherence [15]. These findings press the importance to improve antihypertensive drug adherence to save costs and disease burden. Additionally, understanding the causes and costs associated with HUs in non-adherent patients will aid plan sponsors and policy makers in improving adherence and controlling healthcare costs.

Various studies have been conducted on antihypertensive medications non-adherence in many countries, but complications escorted by non-adherence to these medications reveals necessity for supplemental studies aiming to ascertain the drawbacks and therefore to lower the economic burden of CVDs on the healthcare systems. Medication adherence performs a major role in cost offsets. In general, patients who adhere to their medications exhibit higher treatment costs for apparent reasons. However, on the long run, this behavior leads to decrease in the outlays comparing to non-adherent behaviors, since the inpatients and outpatient visits to the emergency rooms are decreased [16]. Researchers noticed that an extra USD 1 spent on medicines for adherent patients with HT can produce USD 3 to USD 10 in savings on emergency room visits and inpatient hospitalizations [16]. In this study, younger adults, those with the lowest income and those who do not follow regularly with their physicians were more likely to be non-adherent compared with their counterparts. Similarly, prior reports have demonstrated associations between non-adherence and socio-demographic factors such as age and income [17].

This study revealed that adherence generally seemed to increase with age. These findings were consistent with findings from previous studies, in which younger adults were significantly less likely to have high adherence than were older adults [1, 18]. Adherence to prescribed medication matters for the working-age population. Treatment can help prolong productivity in this population and adherence to antihypertensive therapies will improve long-term outcomes [18]. A likely cause may be that younger adults appraise their general health as “excellent”; because many in this age group have not developed physical CVDs symptoms yet; which may stimulate non-adherence behavior [18]. Economically, with the increase of life-span, the health expenditures increase. According to an OECD study, people over 65 counts for 40–50% of health spending in Europe and their per capita costs are 3–5 times higher than those under 65, with propensity to increase over time [19]. Will et al., noticed that hospitalizations were eight times more recurrent in non-adherent elderly hypertensive patients, and the spending with these patients being four times higher compared to adherent patients. The result of non-adherence was USD 41 million, over 3 million for non-adherent hypertensive patients, over a period of 8 years [20]. In addition, Bansilal et al., found that non-adherent elderly patients have higher related medical costs, with USD 719 linked with hospitalizations per patient [21]. Therefore, the higher rate of adherence in elderly in our study provide a promising startup on decreasing healthcare cost related to HU in this population in Lebanon.

In line with findings of other studies [22, 23], our results showed that patients with high income were more likely to be adherent to medications compared to those with low income. Another study showed that non-adherence were 18.51 times more likely to occur in patients with low income than higher income [22]. A systematic review showed that higher household income reduced the risk estimate of non-adherence in 31 of 40 cohorts examined; however, the opposite effect was observed in 8 of 40 cohorts examined [23]. The cause could be described by the incapability of low-income patients to manage their medication cost. Additionally, a study showed that patients with lower household income were more plausibly to be non-adherent and they were also at higher risk for death when they were not adherent to their antihypertensive medication which may suggest that low income and medication non-adherence are essential risk factors for mortality and CVDs, thus increasing healthcare cost [2]. Nevertheless, as the impact of patient’s income status on medication non-adherence was well established in literature [22, 23]. And medication non-adherence was generally associated with higher healthcare costs [2–5]. In consequence, patient’s income status may has effect on healthcare cost. Our results that showed the increase in adherence in patients with higher income reflect an influence on healthcare cost. Governmental interventions to ensure that patients with low income receive recommended care processes could improve control and would increase health care expenditures in the short term. But over the long-term, attaining treatment goals for HT in these patients can be cost-effective and prevent many cardiovascular events, and it might be cost saving to payers.

In this study, those who regularly follow up with their healthcare practitioner were more adherent to their therapy. This result is consistent with the findings of our previous study [24] where the follow-ups and the relationship with physician have been found to have a significant influence on patients’ adherence. This finding is also in line with that of a study, which reported that the highest rate of adherence was associated with physicians’ appointment keeping of patients [24]. Similarly, in a systematic review, Rabbia et al. [25] emphasized the importance of the physicians’ recommendations to enhance the adherence to the treatment by the patients [25]. Notably, other study showed that higher adjusted odds of non-adherence were observed in participants who did not regularly visit a primary health-care provider (AOR = 2.74, 95% CI = 1.09–6.88) [25]. On this basis, we hypothesized that regular referral of patients to their physicians provides a great opportunity for improving their treatment adherence and thus decreasing healthcare cost on long-term.

This study does have limitations. Even though, hospitalization data from MoPH accounts for patients from all governorates, our sample size, used to assess adherence, were collected from Beirut and mount-Lebanon only. While our estimate takes into account directly measured inputs at the national level, it was designed to be conservative in its estimate of non-adherence rate. The self-reported nature of the survey has the disadvantages of recall bias and eliciting only socially acceptable responses and hence, may lead to overestimation of adherence rate. Despite those limitations, this study has strengths as well; the first strength of this study was related to the usage of the MoPH hospitalization data. It pioneered in estimating the cost of hospitalization for HU in non-adherent hypertensive patients. In addition, adherence to antihypertensive medications was measured by using the DAI adherence scale and data were collected by face to face interview with patients; which help to have more complete and accurate information.

## Conclusion

This study was an important first step in evaluating the economic impact of non-adherence to antihypertensive medications in Lebanon. Poor adherence was associated with increase hospitalization cost for HUs. This information may help policy makers develop healthcare plans to minimize economic burden on health system. Decision-makers at all levels need to appraise the adherence to antihypertensive therapies, identify factors that influence it and articulate policies that will achieve better adherence to these therapies. This will help attain treatment goals for HT and prevent potentially preventable hospitalizations, which might be cost saving to government and payers.

## Data Availability

All data generated or analyzed during this study are included in this published article.

## Abbreviations

AHA: American Heart Association
BP: Blood pressure
CSC: Civil servants cooperative
CVD: Cardiovascular diseases
DAI: Drug attitude inventory
HU: Hypertensive urgency
HT: Hypertension
IMS: Intercontinental marketing statistics
GDP: Gross domestic product
LBP: Lebanese pound
MoPH: Ministry of Public Health
NSSF: National Social Security Funds
OR: Odds ratios
SPSS: Statistical package for the social sciences
